# New Antimicrobial Resistance Strategies: An Adaptive Resistance Network Conferring Reduced Glycopeptide Susceptibility in VISA

**DOI:** 10.3390/antibiotics12040783

**Published:** 2023-04-19

**Authors:** Elvira Aguglia, Eleonora Chines, Stefania Stefani, Viviana Cafiso

**Affiliations:** Department of Biomedical and Biotechnological Sciences, University of Catania, 95123 Catania, Italy; elvira.aguglia@gmail.com (E.A.); eleonorac50@gmail.com (E.C.); stefanis@unict.it (S.S.)

**Keywords:** VISA, CA-MRSA, omics, reduced glycopeptide susceptibility, adaptive resistance pathways

## Abstract

**Background**: Vancomycin-intermediate *Staphylococcus aureus* (VISA) emerges typically in the healthcare-associated methicillin-resistant *S. aureus* and more rarely in community-acquired *S. aureus* (CA-MRSA). VISA is a serious concern for public health due to its association with persistent infections, the failure of vancomycin treatment, and poor clinical outcomes. Currently, the burden of VISA is somewhat high, even though vancomycin is the mainstay treatment for severe MRSA infections. The molecular mechanisms of reduced glycopeptide susceptibility in *S. aureus* are constantly under investigation but have still not yet been fully characterized. **Methods:** Our goal was to investigate the reduced glycopeptide susceptibility mechanisms emerging in a VISA CA-MRSA versus its vancomycin-susceptible (VSSA) CA-MRSA parents in a hospitalized patient undergoing glycopeptide treatment. Comparative integrated omics, Illumina MiSeq whole-genome sequencing (WGS), RNA-Seq, and bioinformatics were performed. **Results:** Through a comparison of VISA CA-MRSA vs. its VSSA CA-MRSA parent, mutational and transcriptomic adaptations were found in a pool of genes involved, directly or indirectly, in the biosynthesis of the glycopeptide target conferring or supporting the VISA phenotype, and its cross-resistance with daptomycin. This pool included key genes responsible for the biosynthesis of the peptidoglycan precursors, i.e., D-Ala, the D-Ala-D-Ala dipeptide termini of the pentapeptide, and its incorporation in the nascent pentapeptide, as key targets of the glycopeptide resistance. Furthermore, accessory glycopeptide-target genes involved in the pathways corroborated the key adaptations, and thus, supported the acquisition of the VISA phenotype i.e., transporters, nucleotide metabolism genes, and transcriptional regulators. Finally, transcriptional changes were also found in computationally predicted cis-acting small antisense RNA triggering genes related both to the key or accessory adaptive pathways. **Conclusion:** Our investigation describes an adaptive resistance pathway acquired under antimicrobial therapy conferring reduced glycopeptide susceptibility in a VISA CA-MRSA due to a comprehensive network of mutational and transcriptional adaptations in genes involved in pathways responsible for the biosynthesis of glycopeptide’s target or supporters of the key resistance path.

## 1. Introduction

Glycopeptides are one of the mainstays in the treatment of severe MRSA infections, whilst daptomycin, linezolid, and tigecycline remain the few available last-resort therapeutic options. MRSA can develop increased resistance to glycopeptides, determining the onset of vancomycin-intermediate resistant *S. aureus* (VISA) (MIC = 4–8 mg/L) or heterogenous VISA (hVISA) (MIC ≤ 2 mg/L), which is a mixed cell population—derived originally from a single colony—in which most of the bacterial population has little or no resistance to vancomycin (MIC ≤ 2 mg/L) and a subpopulation is resistant to the antibiotic at the level of VISA (MIC ≥ 4 mg/L) [1]. 

Nowadays, the global emergence of VISA is relatively high, although VISA represents a global challenge for its ability to create persistent infections, the failure of vancomycin treatment, and poor clinical outcomes.

Previous findings reported that hVISA/VISA strains have a thicker cell wall, reduced peptidoglycan cross-linking, and excess free D-Ala-D-Ala residues acting as a decoy for vancomycin’s targets within the cell wall. In addition, D-Ala-D-Ala-bound vancomycin accumulates at the cell wall, limiting the further diffusion of vancomycin [2,3,4]. 

Several studies have shown that VISA with higher levels of vancomycin resistance is less stable, due to the reduced growth and significant fitness costs associated with the mutations leading to a VISA phenotype, and often revert to lower levels of resistance associated with hVISA or to a full vancomycin susceptibility [1,5,6].

At the molecular level, the VISA phenotype was characterized by differentially regulated cell wall biosynthesis and stimulatory pathways [7,8,9,10], the reduced cross-linking of peptidoglycan, the decreased autolytic activity of the enzymes responsible for cell wall turnover [2,11,12,13,14,15,16], altered surface proteins, agr-system dysfunctions, and changes in the growth features [2,17,18,19,20,21]. Frequently, VISA emerges from an hVISA [11,22,23], even though the molecular mechanisms triggering the development of the hVISA/VISA have not been completely defined. There is evidence supporting that the development of hVISA is an epigenetic process rather than one based on gene mutation [24,25]. Interestingly, reduced susceptibility to glycopeptide antibiotics has been associated with increased susceptibility to daptomycin [26,27] and beta-lactams [28,29]. 

Different genes, mutations, and/or transcriptional dysregulation have been reported to be associated with the vancomycin intermediate phenotype [30,31]. They include: (i) *gra*RS (two-component regulatory systems) involved in the regulation of cell wall biosynthesis, the capsule biosynthesis operon, the *dlt* operon and the *mpr*F*/fmtC* genes linked to teichoic acid alanylation and alterations of the cell wall’s charge, global regulators -i.e., rot (repressor of toxins) and agr (accessory gene regulator)-; (ii) *wal*KR (a two-component regulatory systems) increasing capsule synthesis, cell wall thickness, and reducing autolysis; (iii) *vra*SR (a two-component gene regulatory system) regulating up to 40 cell wall synthesis genes and required for producing cell wall derivatives such as D-Ala-D-Ala and (iv) *rpo*B encoding the DNA-dependent RNA polymerase β–subunit commonly associated with increased resistance to vancomycin but also with prolonged propagation time and increased cell wall thickness [31,32,33,34,35,36,37,38,39,40,41,42,43].

Here, our goal was to delineate the network of acquired genomic and transcriptomic changes, directly or indirectly, conferring and/or supporting the glycopeptide reduced susceptibility mechanisms in a VISA CA-MRSA already evaluated for its virulence and high fitness costs.

## 2. Results 

One pair of isogenic *S. aureus* strains of a VISA DAP-R CA-MRSA (1-R) (USA-400, ST-1, spatype-t127, agr-III, SCCmecIVa, PVL-positive) and its VSSA DAP-S CA-MRSA parent (1-S) was collected from a hospitalized patient, as previously described [44,45]. The genomic characterizations, the AMR (antimicrobial resistance) profiles, the fitness costs -in terms of growth retardation and a longer lag growth phase-, and the compensatory adaptations (decreased virulence, a lower transcriptional rate in the essential primary metabolic pathways) were previously described [44,45].

### 2.1. VISA-Related nsSNPs 

**Figure 1:**
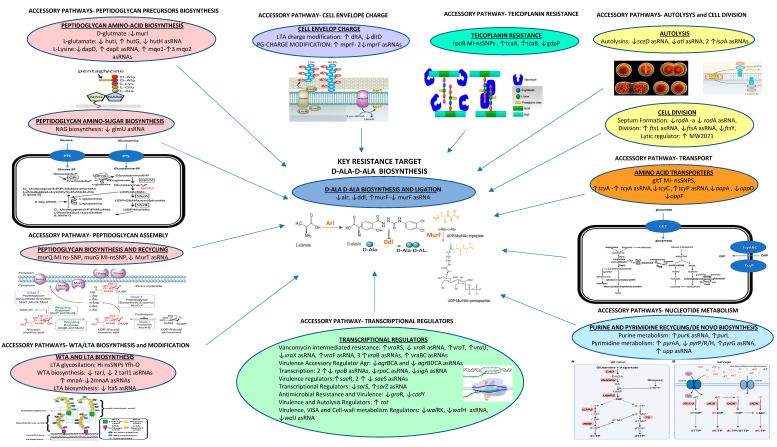
Schematic representation of the affected pathways in VISA CA-MRSA vs. the VSSA CA-MRSA parent.

nsSNPs (non-synonymous SNPs) were found in several accessory genes related to glycopeptide’s targets. In particular, a unique High Impact (HI)-nsSNP was found in MW1125 encoding a YfhO-family protein (lipoteichoic acid glycosylation), whilst Moderate Impact (MI)-nsSNPs were recorded in *mur*Q (N-acetylmuramic acid (NAM) or MurNAc biosynthesis), in *mur*G (biosynthesis of the lipid II PG substrate that penicillin-binding proteins (PBPs) polymerize and cross-link into the cell wall), in *dap*A (L-lysine biosynthesis via diaminopimelic acid), in *glt*T (proton/sodium-glutamate-lactate symport), in *mpr*F (L-lysinylation of the phosphatidylglycerol), in *rpo*B (RNA polymerase β subunit), in the transcriptional regulator *vra*T, and in *gdp*P (c-di-AMP phosphodiesterase) (Table 1) (Figure 1). 

### 2.2. Comparative Transcriptomics 

Gene Ontology (GO) and KEGG (Kyoto Encyclopedia of Genes and Genomes) analyses of the integrated TS (Tru-Seq) and SI (Short-Insert) library RNA-seq datasets revealed crucial dysregulations, both overexpression and underexpression (↓, low rate; ↓↓, medium rate; ↓↓↓, high rate), in key glycopeptide-target related genes involved in the main steps of biosynthesis of the glycopeptide target, and in accessory glycopeptide-target genes implicated in pathways supporting the main paths of resistance. These included the biosynthesis/structure of peptidoglycan and cell wall, the metabolism of amino acid and purine/pyrimidine, the organization of the division septum, transport, and transcriptional regulation. 

Through focusing on the targets directly or indirectly related to the mechanism of action of the glycopeptides, acquired statistically significant differential expression, in terms of DEGs (differentially expressed genes) and computationally predicted cis antisense small RNAs (cis-asRNAs), were found in VISA CA-MRSA versus its VSSA CA-MRSA parent as follows (Table S1) (Figure 1): 

**Key glycopeptide-target related genes** included: (1) biosynthesis of the glycopeptide target (i) biosynthesis of D-alanine: ↓ *alr* (alanine racemase for L-alanine and D-alanine interconversion); (ii) biosynthesis of the D-Ala-D-Ala dipeptide: ↓ *ddl* (D-alanine-D-alanine ligase) and a ↓↓↓ *ddl* asRNA; (iii) incorporation of the D-Ala-D-Ala dipeptide in the murein pentapeptide: ↑ *murF* (UDP-N-acetylmuramoyl-tripeptide-D-alanyl-D-alanine ligase) and a ↓↓↓ *murF* asRNA. 

**Accessory glycopeptide-target genes** included: (a) Cell wall: (1) assembly of peptidoglycan: 2 ↓↓↓ *mur*T cis-asRNAs (alpha-D-isoglutamine formation in the cell wall’s lipid II stem peptide); (2) biosynthesis of peptidoglycan amino sugars: ↓↓↓ *glm*U asRNA (a de novo biosynthetic pathway for UDP-N-acetylglucosamine); (3) biosynthesis of the peptidoglycan amino acid precursor: (i) D-glutamate biosynthesis: ↓*mur*I (glutamate racemase converting the L-glutamate to D-glutamate); (ii) biosynthesis of L-lysine via diaminopimelic acid (DAP): ↓*dap*D, ↑↑↑*dap*E asRNA, ↑*mqo1*, and 3 ↑↑↑/↑↑*mqo2* cis-asRNAs; (iii) biosynthesis of L-glutamate: ↓*hut*I, ↑*hut*G, and ↓↓↓*hut*H asRNA (degradation of L-histidine into L-glutamate); (4) biosynthesis and modification of teichoic acid (TA)/lipoteichoic acid (LTA:P (i) biosynthesis of LTA: ↓↓↓*lta*S asRNA (polymerization of LTA polyglycerol phosphate using phosphatidylglycerol (PG); (ii) biosynthesis of TA: ↓*tar*J (ribulose-5-phosphate reductase, biosynthesis of teichoic acid), ↑*mna*A (UDP-GlcNAc 2-epimerase) and 2 *↓↓↓*/↑↑↑ *mna*A cis-asRNAs, and 2 ↓↓↓ *isp*D *(tar*I1) asRNAs (ribitol-5-phosphate cytidylyltransferase); (iii) modification of LTA: ↑↑*dlt*A (D-alanylation of LTA influencing the net charge of the cell wall and autolysis), and ↓*dlt*D (D-alanylation of LTA); (b) Cell division: (1) autolysis: (i) autolysins: ↓↓↓*sce*D asRNA (lytic transglycosylase that is able to cleave peptidoglycan and affects the clumping and separation of bacterial cells positively regulated by the sigma B factor), ↓↓↓*atl* asRNA (bifunctional N-acetylmuramoyl-L-alanine amidase/endo-beta-N-acetylglucosaminidase), and 2 ↑↑↑ *isa*A cis-asRNAs (putative transglycosylase); (2) septum formation: ↓*rod*A and a ↓↓↓ *rod*A asRNA (cell elongation by interaction between non-essential PBP3 and RodA for the correct localization at the midcell and the insertion of peptidoglycan (PG) at sites other than the septum), ↑↑↑ *fts*L asRNA (recruitment of the putative Lipid II flippase MurJ to the septum driving the peptidoglycan incorporation to the midcell), ↓↓↓*fts*A asRNA (cell division protein tethering FtsZ to the membrane and required for downstream cell division), ↓*fts*Y (involved in targeting and the insertion of nascent membrane proteins into the cytoplasmic membrane), and ↑ MW2071 lytic regulator; (c) Charge of phosphatidylglycerol: (1) modification of phosphatidylglycerol (PG): (i) L-lysinylation of phosphatidylglycerol (PG): ↑*mpr*F (modification of anionic phosphatidylglycerol in positively charged L-lysine phosphatidylglycerol, resulting in a repulsion of the positively charged compound) and 2 ↓↓↓*mpr*F cis-asRNAs; (d) Transporters: (1) AA transporters: ↑↑*tcy*A and a ↑↑↑ *tcy*A asRNA, ↓*tcy*C, ↑↑↑*tcy*P asRNA (DAP and cysteine transporters), ↓*aap*A, ↓*opp*D, and ↓*opp*F; (e) Nucleotide metabolism: (1) metabolism of purine: ↑*pur*L and ↑↑↑ *pur*K asRNA; (2) metabolism of pyrimidine*:* ↑↑↑*pyr*AA, ↓ *pyr*P/R/H, ↑↑↑*pyr*G asRNA, ↑↑ *upp,* and ↓↓↓*upp* asRNA; (f) Resistance to teicoplanin: (1) targets associated with resistance to teicoplanin: ↑*tca*A, ↑↑*tca*B, and ↓*gdp*P; (g) Transcriptional regulators: (1) vancomycin-intermediate resistance: ↑↑*vra*RS and a ↓↓↓ *vra*R asRNA, ↑↑*vra*T, ↑↑*vra*U, ↑↑↑*vra*X asRNA, ↑↑↑*vra*F asRNA, 3 ↑↑↑/↑↑*vra*B cis-asRNAs, and ↑↑ *vra*BC asRNA; (2) virulence accessory regulator (Agr): ↓↓*agr*BCA and ↓↓↓/↓↓ *agr*BDCA cis-asRNA; (3) transcription: 2 ↑↑↑/↓↓↓ *rpo*B cis-asRNAs (RNA polymerase β subunit), ↑↑↑*rpo*C asRNA (RNA polymerase β subunit), and ↓↓↓*sig*A asRNA (RNA polymerase sigma factor, SigA); (4) virulence regulators: ↑↑*sae*R and 2 ↑↑↑/↓↓↓ *sae*S cis-asRNAs; (5) transcriptional master regulators: ↓*sar*S and ↑↑↑*sar*Z asRNA; (6) antimicrobial resistance and virulence: ↓*gra*R (cationic antimicrobial peptide (CAMP) resistance and VISA) and ↓*cod*Y (regulation of metabolic genes); (7) regulators of virulence and autolysis: ↑↑ *rot*; (8) regulators of virulence, VISA, and cell wall metabolism: ↓*wal*RK and ↓↓↓*wal*H asRNA (cell wall turnover and ultimately reduced vancomycin efficacy) and ↓↓↓*wal*J asRNA (YycHI is an activator of YycG). 

A complete representation of the affected pathways both in terms of the nsSNPs and transcriptional dysregulation is shown in Figure 1.

### 2.3. Comparative Analysis of nsSNPs in Intergenic Genomic Regions 

No nsSNPs were detected in intergenic genomic regions—which notoriously contain promoters and regulatory elements—of the statistically significant differentially expressed genes in 1R vs. 1S, as shown in Table S2.

### 2.4. Validation of the Transcriptional Trends 

The validation of the transcriptional trends of the protein-coding genes *mur*F, *hld*, *hla*, *dlt*A, *mpr*F, *spa*, *agr*A, *ica*A, and *sdr*D [45] as well as the MW1303 asRNA (*suc*A) clearly confirmed the overexpression of the marker asRNA found in the RNA-seq transcriptional data output (Figure S1). 

## 3. Discussion

This study explored the mechanism of the onset of reduced glycopeptide susceptibility in a very particular and, at the same time, alarming situation in which an ST-1 CA-MRSA spread in a hospital setting [45]. This event involved the permeation into a hospital of a potentially hypervirulent CA-MRSA that, under high antimicrobial selective pressure, acquired a complex network of multilevel adaptations conferring full reduced glycopeptide and daptomycin susceptibility. This very singular situation selected for a new threatening MRSA lineage that was simultaneously hypervirulent and extensively drug-resistant, emerged through the co-occurrence of genomic, transcriptional, and regulatory adaptations in different cell pathways conferring and maintaining glycopeptide resistance. In addition, our study discovered the presence of a cis-asRNA pool that could confer various unknown regulatory mechanisms to their targets i.e., to control gene expression at multiple levels including transcription, RNA editing, post-transcription, and translation, as previously described [46]. Their presence opens new possibilities in our knowledge about the presence and the activity of the asRNA-regulated transcripts at the level of transcriptional and post-transcriptional downstream effects. 

In this scenario, a new strategy of the mechanism of reduced glycopeptide susceptibility takes shape, which is an adaptive resistance complex of changes in the genes implicated in the pathways, directly or indirectly, triggering the biosynthesis of the glycopeptide-target.

Our data described the multilevel adaptations related to the target of glycopeptide, including nsSNPs, transcriptional variations, and asRNA regulatory mechanisms associated with the acquisition of reduced glycopeptide susceptibility. 

The key trait was represented by an alteration in the D-Ala-D-Ala residue available for the biosynthesis of pentapeptide. Mutations, alterations in the transcriptional rate, and new regulatory mechanisms were found in genes encoding the alanine racemase, converting the L-Ala into D-Ala and into the D-Ala-D-Ala ligase. Interestingly, our study described the presence of an unknown regulatory mechanism of d-alanine-d-alanine ligase production that is in charge of the underexpression of a *ddl* cis-asRNA. This can act both by putative switching on, overexpression, or upregulation as well as by switching off, underexpression, or downregulation of the transcription and/or translation of *ddl*.

The second key feature related to the biosynthesis of the target of glycopeptide was a predicted modification in the rate of the incorporation of the D-Ala-D-Ala into the growing murein pentapeptides via the overexpression of *mur*F coupled to the underexpressed *mur*F cis-asRNA. The overexpression of *mur*F could indicate greater binding of D-Ala-D-Ala to the L-lysine residue and involved a large amount of D-Ala-D-Ala residue being available for trapping and docking glycopeptides, as previously published [4,47]. However, the presence of an underexpressed *mur*F cis-asRNA also indicated the presence of unknown regulatory mechanisms acting on the final step of the synthesis of UDP-N-acetylmuramoyl-pentapeptide that could drastically modify the presence and/or the activity of the MurF UDP-N-acetylmuramoyl-tripeptide-D-alanyl-D-alanine ligase.

Different accessory adaptations occurred in genes involved in the functions indirectly supporting the biosynthesis of the glycopeptide-target. In detail, different genes involved in biosynthesis of the murein precursor i.e., D-glutamate, L-lysine, and amino sugars, showed changes in VISA CA-MRSA. 

Regarding the biosynthesis of D-glutamate, a mutational change (Val232Glu) was found in a gene related to the intracellular transport of glutamate (*glt*T), indicating an upstream adaptation limiting the transport of the glutamate in the cell. This event was associated with a dysregulation in *mur*I, a gene encoding D-glutamate racemase, converting the L-glutamate into D-glutamate, and to a concomitant differential expression of *hut*I, *hut*G, and a *hut*H sRNA, which are involved in the pathway of histidine degradation in L-glutamate. D-glutamate is the second residue of the murein pentapeptide, so these adaptations could determine the poor availability of both L-glutamate and D-glutamate, with obvious implications for the biosynthesis of murein pentapeptide. 

Similarly, for lysine, representing in the L-form of the third amino acid of the murein pentapeptide, mutational and transcription changes were found in de novo biosynthesis of lysine via genes related to diaminopimelate (DAP) (*dap*A, *mqo*1, *dap*D, *dap*E asRNA, *mqo*1, *3 mqo*2 cis-asRNA), (Val280Glu, Ala101Thr), as well as in genes encoding the DAP transporter (*tcy*A, asRNA *tcy*A, *tcy*C). Transcriptional adaptations were also found in other genes encoding amino acid transporters (*aap*A, *opp*D, *opp*F). 

Finally, significant alterations were involved in the genes related to the biosynthesis of purine and pyrimidine indirectly linked to the metabolism of amino acids. The opposite expression trend (overexpression of purine and underexpression of a main pyrimidine) was found in metabolic and regulatory genes for the biosynthesis of purine and pyrimidine (*pur*L, *pur*K, *pyr*R, *pyr*P, *pyr*AA/*car*A, *pyr*H, *pyr*G, *upp*). The role of the metabolic pathways of purine and pyrimidine is controversial, since a transcriptional modification was frequently detected, even if a direct role in an increase in glycopeptide’s MIC has not been found [48]. Our data can support their putative role, since their biosynthesis uses the amino acids aspartate, glutamine, and glycine for purine, as well as glutamate and aspartate for pyrimidine. The observed variation could be associated with a salvage strategy of amino acids via the salvage pathways of purine and pyrimidine or with de novo biosynthesis pathways compensating for the numerous adaptations occurring in the recruitment of amino acids. 

These data could indicate alterations in the access to and trafficking of murein amino acids and their precursors in the bacterial cytoplasm. 

The biosynthesis of amino sugars was another affected pathway. A mutation (Ile156Asn) was found in *mur*Q, N-acetylmuramic acid 6-phosphate etherase (responsible for the conversion of the D-lactyl-MurNAc 6-phosphate to GlcNAc 6-phosphate and D-lactate), and transcriptional changes and a modified regulatory pathway were highlighted for the genes responsible for the biosynthesis of amino sugars via a *glm*U asRNA involved in a de novo biosynthetic pathway for UDP-N-acetylglucosamine (UDP-GlcNAc).

Genes involved in the biosynthesis of peptidoglycan and murein recycling are pathways with variations. Mutations affecting the protein (Ile121Asn) were found in MurG, which is implicated in the assembly of UDP-NAG-NAM-(pentapeptide)-pyrophosphoryl-undecaprenol with N-acetylglucosamine (NAG) to produce Lipid II, the crucial precursors of peptidoglycan, and transcriptional changes occurred in two *mur*T cis-asRNA putatively involved in the regulation of the alpha-D-isoglutamine formation in the cell wall’s Lipid II stem peptide. MurT, together with GatD, determines the glutamate amidation of peptidoglycan, a secondary modification. The MurT–GatD complex is required for the cell’s viability, full resistance to β-lactam antibiotics, and resistance to human lysozymes [7]. Transcriptional and regulatory adaptations were also found in the *mna*A involved in the transformation of UDP-N-acetyl-D-glucosamine (NAG) to UDP-N-acetyl-D-mannosamine. 

Accessory pathways in which adaptations occur at different levels are those of the biosynthesis of autolysin and the regulation of autolysis. The biosynthesis of autolysin is a challenging and intriguing point. All autolysin-related transcripts were found in an antisense orientation as underexpressed cis-asRNA targeting *atl* and *sce*D as well as overexpressed *isa*A. These data have a strong impact, because previously published data indicated that the VISA strain had decreased autolysis via a dysregulation in the *atl* and *sce*D autolysins; however, no previous knowledge of their transcripts’ orientation is known. Here, for the first time, we report the presence of only autolysin-targeting cis-asRNA, indicating new regulatory pathways of an autolysin-coding gene associated with the VISA phenotype, and transcriptional variations in the autolysin-coding genes that were not already known.

Regarding the regulatory genes of autolysis, modifications were found in the genes associated with the biosynthesis and modification of both wall teichoic (WTA) and lipoteichoic acid (LTA). The WTAs are linked to the cell wall and are covalently bound to N-acetylmuramic acid or a terminal D-alanine in the tetrapeptide’s cross-linkage between the N-acetylmuramic acid units of the peptidoglycan layer, whereas the LTAs are anchored in the cytoplasmic membrane by a lipid anchor. Teichoic acids assist in the regulation of cell growth by limiting the ability of the autolysins to break the β-(1–4) bond between N-acetyl glucosamine and N-acetylmuramic acid. A unique HI-nsSNP (Gly75) was found in the gene encoding *yfh*O that is responsible for the glycosylation of LTA, whilst transcriptional changes were found in *tar*J, which catalyzes the NADPH-dependent reduction of D-ribulose 5-phosphate to D-ribitol 5-phosphate in the teichoic acids; and in the regulatory pathways involved in the biosynthesis of WTA and LTA by *tar*I1 cis-asRNA and *lta*S asRNA, which catalyze the polymerization of lipoteichoic acid (LTA) polyglycerol phosphate. These data may agree with previous observations reporting that glycopeptides can act likewise, interfering with cell wall autolysis. In detail, the D-alanine residues of the teichoic acids of *S. aureus* could alter the susceptibility to vancomycin and the activity of autolysins. Some *S. aureus* strains lacking D-alanine esters in the teichoic acids exhibited at least threefold higher sensitivity to glycopeptide antibiotics [49]. Furthermore, the D-alanine mutant could reduce the autolytic activity compared with the wild-type, and the mutant may be more susceptible to the staphylolytic enzyme lysostaphin [49]. Moreover, the teichoic acid D-alanine esters contain positively charged amino groups, while the terminal D-alanine residues of murein peptides contain negatively charged carboxyl groups. 

Furthermore, the increased D-alanylation of LTA due to the overexpression of *dlt*A could determine an over-regulation in the activity of the autolytic systems. Previously data reported that a mutation in *dlt* was more susceptible to glycopeptides, had a low autolysis rate and was resistant to lysozymes. Finally, the direct binding of vancomycin to the D-alanine esters of teichoic acids is an unlikely modification of the charge shared with daptomycin.

The cell division pathway is also involved as an accessory path. Adaptive shifts were found in genes encoding two cell division proteins, i.e., FtsY and RodA, as well as modifications in the regulatory pathways of cell division that can be supposed by the presence of several cell division protein-targeted cis-asRNA (*fts*L, *fts*A, *rod*A *spo*VG, and lytic regulatory protein). 

An additional accessory control point was the charge repulsion due to modification of the cell envelope, conferring a more positive charge, indicated by two MI nsSNPs (Thr345Ala, Leu538Phe) in *mpr*F, and a dysregulation of *dlt*A, as well as *mpr*F and *mpr*F cis-asRNA, which are responsible for the D-alanylation of LTA and the L-lysynilation of phosphatidylglycerol, respectively. Previous data reported that a mutation in *dlt* was more susceptible to glycopeptides, as previously published [50,51], and dysregulated *dlt*A was recovered in DAP-R and hVISA or VISA strains. This trait confers a cross-resistance mechanism to glycopeptides and daptomycin, as previously published [50,51].

Other genes previously associated with glycopeptide resistance showed adaptations. In detail, as previously published, the role of *tca*A genes in teicoplanin resistance is still not fully clarified, whereas the discovery of a c-di-AMP phosphodiesterase GdpP mutation (Ile186Met) and differential expression trends could support the concept that the abnormal c-di-AMP levels could be involved in a complex signaling cascade, blocking the biosynthesis of peptidoglycans and impacting on the tolerance of glycopeptides and β-lactams [52].

Multiple transcriptional changes were found among the genes encoding for transcriptional regulators and their regulatory mechanisms. In addition to the already published dysregulation in the agr locus [45], a higher trend was found in the *vra* gene cluster, *sae* regulators, and *rot*, whilst a lower transcriptional profile was discovered in *wal*RS, *sar*S, and *gra*R. The strong activation of the *vra* gene cluster is a crucial point, since the three-component signal transduction system of VraTSR modulates the expression of the cell wall’s stress stimulon in response to several different cell-wall-active antibiotics. The WalKR in *S. aureus* is, in fact, essential for bacterial viability, regulation of the synthesis of cell walls, and physiological metabolic processes [53]. 

## 4. Conclusions

Our data defined a complex network of adaptations that showed key changes in the processes of D-Ala and D-Ala-D-Ala biosynthesis as well as their incorporation in the nascent pentapeptide, which is the main glycopeptide target, and involved numerous supporting collateral pathways. 

At the biosynthetic level, the first level of adaptation works on the metabolism of peptidoglycan amino acids and amino sugars. This control point influences the metabolism of peptidoglycan’s precursors, acting both on intracellular transport of the pentapeptide amino acid as well as salvage and de novo biosynthesis pathways allowing the recycling of the amino acid’s intracellular reserves, and on the biosynthesis of new amino sugars. 

At the cellular level, there are different regulatory points, such as the assembly of peptidoglycan and murein recycling, influencing the peptidoglycan backbone, the autolytic and cell division process affecting the division process, the thickness of the cell wall, along with the modifications of the cell wall envelope’s charge, affecting the binding of glycopeptide and daptomycin via electrostatic repulsion.

Our investigation describes a new strategy of adaptive antimicrobial resistance related to the biosynthesis of the glycopeptide-target caused by a comprehensive network of pathways accumulating mutational changes and transcriptomic shifts responsible for the emergence and the maintenance of the VISA phenotype.

## 5. Materials and Methods 

### 5.1. Bacterial Strains

One pair of isogenic *S. aureus* strains (1-S and 1-R) of ST-1 (agr-III, delta-hemolysin negative) was recovered from a patient hospitalized in an Italian hospital, as previously described [44,45]. Of note, the second isogenic MRSA (1-R), isolated under teicoplanin therapy, was characterized by additional resistance to the glycopeptides, i.e. vancomycin (MIC 8 mg/L), teicoplanin (MIC 32 mg/L) and dalbavancin (a lipoglycopeptide) (MIC 2 mg/L), and daptomycin (MIC 2 mg/L) according to the EUCAST guidelines 2021 [54]. The antimicrobial susceptibility and molecular characterization were determined previously [44,45].

### 5.2. Whole-Genome Sequencing 

Genomic DNA was extracted using the PureLink Genomic DNA Mini Kit (Invitrogen, Waltham, MA, USA) following the manufacturer’s protocol. The DNA’s quality was evaluated by Qubit, and its concentration was determined by Picogreen (Life Technologies, Carlsbad, CA, USA). Whole-genome sequencing (WGS) was performed using the Illumina Mi-Seq sequencing system, using both a paired-end library with 150 bp reads (400 bp average insert size) and a mate-pair library with 250 bp reads (an average insert size of 8 kb). After generation of the sequence data, raw reads were processed by Fast QC (v0.11.7) to assess the quality of the data, and the Trimmomatic tool (v0.38) was used to remove sequencing adapters for paired-end reads to filter low-quality bases (Q-score < 30) and short reads (<150 bp) as well as mate-pair reads from the process by requiring a minimum base quality of 20 (Phred scale) and a minimum read length of 100 nucleotides to filter out sequences composed only of Ns and to improve the per base score of the mate-pair reads. The total number of PE and MP reads is reported with the estimated coverage in Supplementary Table S1. The trimmed reads were used for downstream analysis [26,45,55].

### 5.3. De Novo Genome Assembly

De novo genome assembly was performed using SPAdes software (v3.12.0). The reads were initially normalized with khmer 1.3, and then they were error-corrected using the Bayesian Hammer utility for SPAdes. Finally, assembly was performed using the recommended parameters for such Illumina data. The SPAdes software produced a contigs file for each sample, and the post-assembly controls and metrics were evaluated using Quast software (v4.6.3) as previously published [26,45,55]. 

### 5.4. Gene Annotation

The assembled contigs were processed using Prokka software (1.14.6) to predict the genes and annotate these sequences using a core set of conserved prokaryotic genes [26,45,55].

### 5.5. Single Nucleotide Variants (SNVs)

For SNVs, genomic resequencing was performed on the paired-end library’s raw reads as already published [27,34]. Briefly, raw reads of Illumina were trimmed by the Trimmomatic tool (v0.38), requiring a minimum base quality of 20 (Phred scale) and a minimum read length of 36 nucleotides. Only trimmed reads were included in the downstream analysis. Each sample was aligned by BWA v. 0.7.5 with *S. aureus* MW2 (BA000033.2) and used as a reference genome. Each .bam file was sorted by Samtools (v.0.1.19), and duplicate reads were marked using the Picard Mark Duplicates utility. Complex variants, SNVs, and indels were detected by Freebayes (v.0.9.14), requiring a minimum mapping quality of 25 (Phred scale) and a minimum base quality of 30. Sequenced reads were properly aligned with the reference genome, with 97.28% for 1-S and 97.29% for 1-R.

To select only the SNVs present in the VISA DAP-R- CA-MRSA, wg nsSNVs were computationally filtered versus those present in the VSSA DAP-S CA-MRSA parent [45]. All non-synonymous SNPs present in the VISA CA-MRSA isolates were confirmed by Sanger sequencing.

### 5.6. Predicted Effects of Whole-Genome Single Nucleotide Polymorphisms (wgSNPs) 

The predicted effects of wgSNP were evaluated using snpEff (v.4.3T). A classification of high (HI), low (LI), moderate (MI), or modified impact (MFI) was assigned according to the criteria previously published [45,56]. In detail for high impact, the variant was assumed to have disruptive impact, probably causing protein truncation, loss of function, or triggering nonsense-mediated decay; low impact was assumed to be mostly harmless or unlikely to change the protein’s behavior; moderate impact involved a non-disruptive variant that might change the protein’s effectiveness; and modified impact usually involved non-coding variants or variants affecting non-coding genes for which predictions are difficult, or there is no evidence of impact [55]. Only wgSNPs with a high (HI) or moderate (MI) effect were considered and subsequently described.

### 5.7. Phylogeny and Genomic Epidemiology 

The whole-genome sequences of the raw data were evaluated by the free tools of the Center for Genomic Epidemiology (CGE, http://www.genomicepidemiology.org) to investigate the genetic and molecular features of the strain-pair. In detail, spaTyper (v1.0) was used to determine the staphylococcal protein A (spa) type of each strain, SCCmecFinder (v1.2) identified the staphylococcal cassette chromosome mec (SCCmec), PlasmidFinder (v2.0) was used for the plasmid search, PHAge Search Tool (PHAST) was used to consider only the prophage regions detected with a completeness score of >90 to detect the prophages, VirulenceFinder (v2.0) was used to identify the virulence factors to define the virulome, and ResFinder (v3.2) was used for detecting the acquired antimicrobial resistance genes [57,58,59,60,61,62,63]. Analyses were performed with the default parameter settings [45]. 

### 5.8. RNA-Seq

#### 5.8.1. RNA-Seq Bacterial Cultures

An aliquot of an overnight culture was diluted 1:50 in 30 mL of brain–heart infusion (BHI) in a sterile 50 mL flask (OD600 nm 0.05) to obtain approximately 5 × 10^5^ CFU/mL of the inoculum for each strain. Cells were grown under shaking at 250 rpm under normal atmospheric conditions at 37 °C and harvested in the exponential growth phase (OD600 0.5, 2 × 10^8^ CFU/mL ~3–4 h). RNA extraction started immediately after cell harvesting to maintain the RNA’s integrity. The cell density was determined by counting the colonies after plating onto Mueller–Hinton (MH) agar and incubation [45]. 

#### 5.8.2. RNA-Seq Libraries

RNA-seq was carried out using the Illumina Mi-seq sequencing platform. To improve the RNA-seq data, two replicates using two different libraries were used, a single-end library with 50 bp reads (SI, short-insert library) and a paired-end read library with 150 bp reads (TS, Tru-Seq library) and an average insert size of 350/400 bp [45]. 

#### 5.8.3. RNA Extraction

Specific RNA extractions for preparation of the Tru-Seq library and the short-insert library were performed according to the specific protocols as a strategy to optimize the collected RNA-seq data, as previously published [26,45,55].

#### 5.8.4. Preparation of the Tru-Seq Library 

The quality of the total RNA was verified using a 2200 TapeStation RNA Screen Tape device (Agilent, Santa Clara, CA, USA), and the concentration was ascertained using an ND-1000 spectrophotometer (NanoDrop, Wilmington, DE, USA). The Agilent TapeStation 2200 system, an automated instrument for nucleic acid gel electrophoresis, assigned RNA integrity number (RIN) values ranging from 1 to 10, with 10 being the highest quality. Only samples with preserved 16S and 23S peaks and RIN values > 8 were used for construction of the library. RIN values > 8 indicated intact high-quality RNA samples for downstream applications, as previously published [26,55]. Ribosomal RNA was removed using the Bacteria Ribo-Zero rRNA Removal Kit from 2 μg of RNA. The depleted RNA was used for the Illumina Tru-Seq RNA stranded kit without PolyA enrichment. The obtained libraries were evaluated with high-sensitivity D1000 screening tape (Agilent Tape Station 2200), and the indexed libraries were quantified with the ABI9700 qPCR instrument using the KAPA Library Quantification Kit in triplicate, according to the manufacturer’s protocol (Kapa Biosystems, Woburn, MA, USA). From the pooled library, final concentrations of 2 nm were used for sequencing with a 150 PE read sequencing module [26,45,55].

#### 5.8.5. Preparation of the Short-Insert Library 

After ribosomal depletion, sequencing libraries were created using the Illumina mRNA-seq sample preparation kit following the supplier’s instructions, except that the total RNA was not fragmented, and the double-stranded cDNA was size-selected (100–400 bp) to maximize the recovery of small RNAs. The prepared libraries were evaluated with high-sensitivity D1000 screening tape (Agilent Tape Station 2200), as described for the TS Library. The indexed libraries were quantified in triplicate with the ABI7900 qPCR instrument using the KAPA Library Quantification Kit, according to the manufacturer’s protocol (Kapa Biosystems, Woburn, MA, USA). From the pooled library, 5 μL at a final concentration of 4 nM wewasre used for MiSeq sequencing with a single-end stranded library with the reads of a 50-bp sequencing module [26,45,55].

#### 5.8.6. Post-Processing of the Tru-Seq Library’s Raw Reads 

After generation of the sequencing data, the raw reads were processed using FastQC (v.0.11.2) to assess the quality of the data. The sequenced reads were then trimmed using Trimmomatic (v.0.33.2) to remove only the sequencing adapters for PE reads. A minimum base quality of 15 (Phred scale) over a four-base sliding window was required. Only sequences with a length above 36 nucleotides were included in the downstream analysis; likewise, only trimmed reads were included in the downstream analysis [26,45,55].

#### 5.8.7. Post-Processing of the Short-Insert Library’s Raw Reads 

After generation of the sequencing data, the raw reads were processed using FastQC (v.0.11.2) to assess the quality of the data. The reads were then trimmed using Trimmomatic (v.0.33.2) to remove the sequencing adapters for single-end reads, requiring a minimum base quality of 15 (Phred scale) and a minimum read length of 15 nucleotides. Only trimmed reads were included in the downstream analysis [26,45,55].

#### 5.8.8. Analysis of the Tru-Seq and Short-Insert Reads 

The TS and SI RNA-seq reads were annotated on *S. aureus* MW2 (BA000033.2), which was used as the RefGen, as well as the transcripts assembled and quantified using Rockhopper (v.2.03) [26,56]. The analyses were run using the default parameter settings with verbose output to obtain the expression data. Rockhopper normalized the read counts for each sample using the expression level of the genes in the upper quartile. Based on the *p*-values calculated according to the approach of Anders and Huber, differentially expressed genes (DEGs) were assigned by computing q-values ≤ 0.01 based on Benjamini–Hochberg correction with a false discovery rate of <1%. In addition, Rockhopper is a versatile tool using biological replicates when available and surrogate replicates when biological replicates for two different conditions are unavailable, considering the two conditions under investigation as surrogate replicates for each other [26,45,55].

### 5.9. DAVID Enrichment Analysis

The online tool DAVID (v.6.8) (Database for Annotation, Visualization, and Integrated Discovery) (https://david.ncifcrf.gov/) was used to detect the affected pathways among the DEGs. The gene lists of the strain–pair, grouped according to over- and underexpressed genes, were uploaded as the official gene symbols of the *S. aureus* MW2 reference genome, automatically selecting the list type (gene list) of *S. aureus*. The functional annotation chart was obtained using an EASE score threshold of ≤0.5 and a minimum number of four genes. DAVID’s functional categories were investigated by the KEGG pathway and PANTHER classification system [64], and grouped in refined annotation clusters of the same genes [45].

### 5.10. Real-Time qPCR Validation

To validate the RNA-seq data, real-time qPCRs for a set of characteristic transcripts, namely *mur*F, *dlt*A, *mpr*F, *hld*, *hla*, *spa*, *agr*A, *ica*A, and *sdr*D, were carried out in the same RNA-seq growth phase [45,54,65]. 

For validation of the expression of sRNA, MW1303 asRNA (*suc*A), encoding the alpha-ketoglutarate dehydrogenase, was an essential metabolic gene that was selected for validation because of its high level of overexpression in 1-R versus 1-S [66]. The genome position and orientation of the MW1303 asRNA is reported in Table 2.

## Figures and Tables

**Table 1:** nsSNPs in targets with reduced glycopeptide susceptibility in VISA CA-MRSA vs. VSSA CA-MRSA.

Gene	Function	Product	nsSNPs AA Changes
**Peptidoglycan Assembly**			
MW0165	N-acetyl muramic acid recycling	N-acetylmuramic acid 6-phosphate etherase MurQ	**MI-nsSNPs** Ile156Asn
MW1307	Lipid II PG substrate for biosynthesis of peptidoglycan	UDP-NAG-NAM-(pentapeptide) pyrophosphoryl-undecaprenol N-acetylglucosamine transferase MurG	MI-nsSNPs Ile121Asn
**Biosynthesis of Peptidoglycan AA**			
MW2286	Intermediate metabolic compound of lysine biosynthesis	Malate:quinone oxidoreductase 1 Mqo1	**MI-nsSNPs** Val280Glu
MW1283	L-lysine biosynthesis via the DAP pathway	Dihydrodipicolinate synthase DapA	**MI-nsSNPs** Ala101Thr
**Biosynthesis of lipotheicoic acid**			
MW1125	LTA glycosylation	YfhO membrane protein	**HI-nsSNPs** Gly75 *
**Charge of the Cell Envelope**			
MW1247	Charge of the cell envelope	Phosphatidylglycerol lysyl-transferase MprF	**MI-nsSNPs** Thr345Ala Leu538Phe
**Regulators of Vancomycin-Intermediate Resistance**			
MW1826	Methicillin resistance and activation of vraSR TCRS	VraT	**MI-nsSNPs** Ala59Glu
**Glycopeptide-Β-Lactams Resistant-Related Genes**			
MW0014	β-lactams and/or glycopeptide cross-resistance	GdpP	**MI-nsSNPs** Ile186Met
**Metabolic Substrate Transporters**			
MW2304	Proton/sodium-glutamate symport	GltT	MI-nsSNPs Val232Glu

Legend: * stop codon.

**Table 2:** Genome position and orientation of the MW1303 asRNA.

CA-MRSA MW2 Locus Tag	sRNA	RefGen Position (nt)		sRNA Size (bp)	Library	RPKM 1R	RPKM 1S
MW1303	Predicted antisense small RNA	1423626	1423663	37	SI	969	0

## Data Availability

The genomic and transcriptomic reads were deposited in the genome database of the National Center for Biotechnology Information (NCBI) in the Sequence Read Archive (SRA) under the BioProject PRJNA860577 with the biosample’s genomic paired-end and RNA-seq raw sequences n° SAMN29849119 and SAMN29849120, and with biosample’s genomic mate-pair draft sequences, n° SAMN30428603 and SAMN30428604.

## Supplementary Materials

The following supporting information can be downloaded at: https://www.mdpi.com/article/10.3390/antibiotics12040783/s1. Figure S1: Validation of sucA asRNA. Table S1: DEGs and cis-asRNA in targets putatively related to glycopeptide resistance in VISA CA-MRSA. Table S2: Comparative analysis of the nsSNPs in the intergenic genomic regions in 1R vs. 1S.

## References

[1] Howden B.P., Davies J.K., Johnson P.D.R., Stinear T.P., Grayson M.L. (2010). Reduced vancomycin susceptibility in *Staphylococcus aureus*, including vancomycin-intermediate and heterogeneous vancomycin-intermediate strains: Resistance mechanisms, laboratory detection, and clinical implications. Clin. Microbiol. Rev..

[2] Cui L., Ma X., Sato K., Okuma K., Tenover F.C., Mamizuka E.M., Gemmell C.G., Kim M.N., Ploy M.C., El Solh N. (2003). Cell wall thickening is a common feature of vancomycin resistance in *Staphylococcus aureus*. J. Clin. Microbiol..

[3] Cui L., Iwamoto A., Lian J.Q., Neoh H.M., Maruyama T., Horikawa Y., Hiramatsu K. (2006). Novel mechanism of antibiotic resistance originating in vancomycin-Intermediate *Staphylococcus aureus*. Antimicrob. Agents. Chemother..

[4] Pereira P.M., Filipe S.R., Tomasz A., Pinho M.G. (2007). Fluorescence ratio imaging microscopy shows decreased access of vancomycin to cell wall synthetic sites in vancomycin-resistant *Staphylococcus aureus*. Antimicrob. Agents. Chemother..

[5] Gardete S., Kim C., Hartmann B.M., Mwangi M., Roux C.M., Dunman P.M., Chambers H.F., Tomasz A. (2012). Genetic pathway in acquisition and loss of vancomycin resistance in a methicillin resistant *Staphylococcus aureus* (MRSA) strain of clonal type USA300. PLOS Pathog..

[6] Boyle-Vavra S., Berke S.K., Lee J.C., Daum R.S. (2000). Reversion of the glycopeptide resistance phenotype in *Staphylococcus aureus* clinical isolates. Antimicrob. Agents. Chemother..

[7] Boyle-Vavra S., Carey R.B., Daum R.S. (2001). Development of vancomycin and lysostaphin resistance in a methicillin-resistant *Staphylococcus aureus* isolate. J. Antimicrob. Chemother..

[8] Daum R.S., Gupta S., Sabbagh R., Milewski W.M. (1992). Characterization of *Staphylococcus aureus* isolates with decreased susceptibility to vancomycin and teicoplanin: Isolation and purification of a constitutively produced protein associated with decreased susceptibility. J. Infect. Dis..

[9] Hanaki H., Kuwahara-Arai K., Boyle-Vavra S., Daum R., Labischinski H., Hiramatsu K. (1998). Activated cell-wall synthesis is associated with vancomycin resistance in methicillin-resistant *Staphylococcus aureus* clinical strains Mu3 and Mu50. J. Antimicrob. Chemother..

[10] Moreira B., Boyle-Vavra S., Daum R.S. (1997). Increased production of penicillin-binding protein 2, increased detection of other penicillin-binding proteins, and decreased coagulase activity associated with glycopeptide resistance in *Staphylococcus aureus*. Antimicrob. Agents Chemother..

[11] Howden B.P., Johnson P.D., Ward P.B., Stinear T.P., Davies J.K. (2006). Isolates with low-level vancomycin resistance associated with persistent methicillin-resistant *Staphylococcus aureus* bacteremia. Antimicrob. Agents Chemother..

[12] Boyle-Vavra S., Challapalli M., Daum R.S. (2003). Resistance to autolysis in vancomycin-selected *Staphylococcus aureus* isolates precedes vancomycin-intermediate resistance. Antimicrob. Agents Chemother..

[13] Boyle-Vavra S., Labischinski H., Ebert C.C., Ehlert K., Daum R.S. (2001). A spectrum of changes occurs in peptidoglycan composition of glycopeptide-intermediate clinical *Staphylococcus aureus* isolates. Antimicrob. Agents Chemother..

[14] Renzoni A., Barras C., François P., Charbonnier Y., Huggler E., Garzoni C., Kelley W.L., Majcherczyk P., Schrenzel J., Lew D.P. (2006). Transcriptomic and functional analysis of an autolysis-deficient, teicoplanin-resistant derivative of methicillin-resistant *Staphylococcus aureus*. Antimicrob. Agents Chemother..

[15] Scherl A., François P., Charbonnier Y., Deshusses J.M., Koessler T., Huyghe A., Bento M., Stahl-Zeng J., Fischer A., Masselot A. (2006). Exploring glycopeptide-resistance in Staphylococcus aureus: A combined proteomics and transcriptomics ap-proach for the identification of resistance-related markers. BMC Genom..

[16] Vaudaux P., Francois P., Berger-Bächi B., Lew D.P. (2001). In vivo emergence of subpopulations expressing teicoplanin or vancomycin resistance phenotypes in a glycopeptide-susceptible, methicillin-resistant strain of *Staphylococcus aureus*. J. Antimicrob. Chemother..

[17] Koehl J.L., Muthaiyan A., Jayaswal R.K., Ehlert K., Labischinski H., Wilkinson B.J. (2004). Cell wall composition and decreased autolytic activity and lysostaphin susceptibility of glycopeptide-intermediate *Staphylococcus aureus*. Antimicrob. Agents Chemother..

[18] McCallum N., Karauzum H., Getzmann R., Bischoff M., Majcherczyk P., Berger-Bächi B., Landmann R. (2006). In vivo survival of teicoplanin-resistant *Staphylococcus aureus* and fitness cost of teicoplanin resistance. Antimicrob. Agents Chemother..

[19] Muthaiyan A., Jayaswal R.K., Wilkinson B.J. (2004). Intact mutS in laboratory-derived and clinical glycopeptide-intermediate *Staphylococcus aureus* strains. Antimicrob. Agents Chemother..

[20] Pfeltz R.F., Singh V.K., Schmidt J.L., Batten M.A., Baranyk C.S., Nadakavukaren M.J., Jayaswal R.K., Wilkinson B.J. (2000). Characterization of passage-selected vancomycin-resistant *Staphylococcus aureus* strains of diverse parental backgrounds. Antimicrob. Agents Chemother..

[21] Sakoulas G., Eliopoulos G.M., Moellering R.C., Wennersten C., Venkataraman L., Novick R.P., Gold H.S. (2002). Accessory gene regulator (agr) locus in geographically diverse *Staphylococcus aureus* isolates with reduced susceptibility to vancomycin. Antimicrob. Agents Chemother..

[22] Mwangi M.M., Wu S.W., Zhou Y., Sieradzki K., de Lencastre H., Richardson P., Bruce D., Rubin E., Myers E., Siggia E.D. (2007). Tracking the in vivo evolution of multidrug resistance in *Staphylococcus aureus* by whole-genome sequencing. Proc. Natl. Acad. Sci. USA.

[23] Sieradzki K., Roberts R.B., Haber S.W., Tomasz A. (1999). The Development of vancomycin resistance in a patient with methicillin-resistant *Staphylococcus aureus* infection. N. Engl. J. Med..

[24] Roch M., Clair P., Renzoni A., Reverdy M.E., Dauwalder O., Bes M., Martra A., Freydière A.M., Laurent F., Reix P. (2014). Exposure of *Staphylococcus aureus* to subinhibitory concentrations of β-Lactam antibiotics induces heteroge-neous vancomycin-intermediate *Staphylococcus aureus*. Antimicrob. Agents Chemother..

[25] Haaber J., Friberg C., McCreary M., Lin R., Cohen S.N., Ingmer H. (2015). Reversible antibiotic tolerance induced in *Staphylococcus aureus* by concurrent drug exposure. mBio.

[26] Cafiso V., Stracquadanio S., Lo Verde F., De Guidi I., Zega A., Pigola G., Stefani S. (2020). Genomic and Long-Term Transcriptomic Imprints Related to the Daptomycin Mechanism of Action Occurring in Daptomycin- and Methicillin-Resistant *Staphylococcus aureus* Under Daptomycin Exposure. Front. Microbiol..

[27] Mishra N.N., McKinnell J., Yeaman M.R., Rubio A., Nast C.C., Chen L., Kreiswirth B.N., Bayer A.S. (2011). In vitro cross-resistance to daptomycin and host defense cationic antimicrobial peptides in clinical methicillin-resistant *Staphylococcus aureus* isolates. Antimicrob. Agents Chemother..

[28] Steinkraus G., White R., Friedrich L. (2007). Vancomycin MIC creep in non-vancomycin-intermediate *Staphylococcus aureus* (VISA), vancomycin-susceptible clinical methicillin-resistant *S. aureus* (MRSA) blood isolates from 2001–05. J. Antimicrob. Chemother..

[29] Howe R.A., Monk A., Wootton M., Walsh T.R., Enright M.C. (2004). Vancomycin susceptibility within methicillin-resistant *Staphylococcus aureus* lineages. Emerg. Infect. Dis..

[30] Meehl M., Herbert S., Götz F., Cheung A. (2007). Interaction of the GraRS two-component system with the VraFG ABC transporter to support vancomycin-intermediate resistance in *Staphylococcus aureus*. Antimicrob. Agents Chemother..

[31] McEvoy C.R.E., Tsuji B., Gao W., Seemann T., Porter J.L., Doig K., Ngo D., Howden B.P., Stinear T.P. (2013). Decreased vancomycin susceptibility in *Staphylococcus aureus* caused by IS256 tempering of *wal*KR expression. Antimicrob. Agents Chemother..

[32] Herbert S., Bera A., Nerz C., Kraus D., Peschel A., Goerke C., Meehl M., Cheung A., Götz F. (2007). Molecular basis of resistance to muramidase and cationic antimicrobial peptide activity of lysozyme in staphylococci. PLoS Pathog..

[33] Howden B.P., Smith D.J., Mansell A., Johnson P.D., Ward P.B., Stinear T.P., Davies J.K. (2008). Different bacterial gene expression patterns and attenuated host immune responses are associated with the evolution of low-level vancomycin resistance during persistent methicillin-resistant *Staphylococcus aureus* bacteraemia. BMC Microbiol..

[34] Nishi H., Komatsuzawa H., Fujiwara T., McCallum N., Sugai M. (2004). Reduced content of lysyl-phosphatidylglycerol in the cytoplasmic membrane affects susceptibility to moenomycin, as well as vancomycin, gentamicin, and antimicrobial peptides, in *Staphylococcus aureus*. Antimicrob. Agents Chemother..

[35] Peschel A., Otto M., Jack R.W., Kalbacher H., Jung G., Götz F. (1999). Inactivation of the *dlt* operon in *Staphylococcus aureus* confers sensitivity to defensins, protegrins, and other antimicrobial peptides. J. Biol. Chem..

[36] Ruzin A., Severin A., Moghazeh S.L., Etienne J., Bradford P.A., Projan S.J., Shlaes D.M. (2003). Inactivation of *mpr*F affects vancomycin susceptibility in *Staphylococcus aureus*. Biochim. Biophys. Acta.

[37] McAleese F., Wu S.W., Sieradzki K., Dunman P., Murphy E., Projan S., Tomasz A. (2006). Overexpression of genes of the cell wall stimulon in clinical isolates of *Staphylococcus aureus* exhibiting vancomycin-intermediate-*S. aureus*-type resistance to vancomycin. J. Bacteriol..

[38] Utaida S., Dunman P.M., Macapagal D., Murphy E., Projan S.J., Singh V.K., Jayaswal R.K., Wilkinson B.J. (2003). Genome-wide transcriptional profiling of the response of *Staphylococcus aureus* to cell-wall-active antibiotics reveals a cell-wall-stress stimulon. Microbiology.

[39] Gardete S., Wu S., Gill S., Tomasz A. (2006). Role of VraSR in antibiotic resistance and antibiotic-induced stress response in *Staphylococcus aureus*. Antimicrob. Agents Chemother..

[40] Howden B.P., Stinear T.P., Allen D.L., Johnson P.D., Ward P.B., Davies J.K. (2008). Genomic analysis reveals a point mutation in the two-component sensor gene *gra*S that leads to intermediate vancomycin resistance in clinical *Staphylococcus aureus*. Antimicrob. Agents Chemother..

[41] Kuroda M., Kuroda H., Oshima T., Takeuchi F., Mori H., Hiramatsu K. (2003). Two-component system VraSR positively modulates the regulation of cell-wall biosynthesis pathway in *Staphylococcus aureus*. Mol. Microbiol..

[42] Cui L., Isii T., Fukuda M., Ochiai T., Neoh H.M., Camargo I.L., Watanabe Y., Shoji M., Hishinuma T., Hiramatsu K. (2010). An RpoB mutation confers dual heteroresistance to daptomycin and vancomycin in *Staphylococcus aureus*. Antimicrob. Agents Chemother..

[43] Matsuo M., Hishinuma T., Katayama Y., Cui L., Kapi M., Hiramatsu K. (2011). Mutation of RNA polymerase beta subunit (*rpo*B) promotes hVISA to-VISA phenotypic conversion of strain Mu3. Antimicrob. Agents Chemother..

[44] Capone A., Cafiso V., Campanile F., Parisi G., Mariani B., Petrosillo N., Stefani S. (2016). In Vivo development of daptomycin resistance in vancomycin-susceptible methicillin-resistant *Staphylococcus aureus* severe infections previously treated with glycopeptides. Eur. J. Clin. Microbiol. Infect. Dis..

[45] Salemi R., Zega A., Aguglia E., Lo Verde F., Pigola G., Stefani S., Cafiso V. (2022). Balancing the Virulence and Antimicrobial Resistance in VISA DAP-R CA-MRSA Superbug. Antibiotics.

[46] Saberi F., Kamali M., Najafi A., Yazdanparast A., Moghaddam M.M. (2016). Natural antisense RNAs as mRNA regulatory elements in bacteria: A review on function and applications. Cell. Mol. Biol. Lett..

[47] Peschel A., Vuong C., Otto M., Götz F. (2000). The D-alanine residues of *Staphylococcus aureus* teichoic acids alter the susceptibility to vancomycin and the activity of autolytic enzymes. Antimicrob. Agents Chemother..

[48] Paige M., Fox M.W.C., Gordon L.A. (2007). Lack of Relationship between Purine Biosynthesis and Vancomycin Resistance in *Staphylococcus aureus*: A Cautionary Tale for Microarray Interpretation. Antimicrob. Agents Chemother..

[49] Gillen A.L., Conrad J., Cargill M. (2015). The Genesis and Emergence of Community-Associated Methicillin-Resistant *Staphylococcus aureus* (CA-MRSA): An Example of Evolution in Action?. Answ. Res. J..

[50] Cafiso V., Bertuccio T., Spina D., Purrello S., Campanile F., Di Pietro C., Purrello M., Stefani S. (2012). Modulating activity of vancomycin and daptomycin on the expression of autolysis cell-wall turnover and membrane charge genes in hVISA and VISA strains. PLoS ONE..

[51] Griffiths J.M., O’Neill A.J. (2012). Loss of function of the *gdp*P protein leads to joint β-lactam/glycopeptide tolerance in *Staphylococcus aureus*. Antimicrob. Agents Chemother..

[52] Cafiso V., Bertuccio T., Purrello S., Campanile F., Mammina C., Sartor A., Raglio A., Stefani S. (2014). *dlt*A overexpression: A strain-independent keystone of daptomycin resistance in methicillin-resistant *Staphylococcus aureus*. Int. J. Antimicrob. Agents.

[53] Almeida S.T., Paulo A.C., Babo J., Borralho J., Figueiredo C., Gonçalves B., Lança J., Louro M., Morais H., Queiroz J. (2021). Absence of methicillin-resistant *Staphylococcus aureus* colonization among immunocompetent healthy adults: Insights from a longitudinal study. PLoS ONE.

[54] EUCAST. https://www.eucast.org/ecast_news/news_sileview/?tx_ttnews%5Btt_news%5D=459cHash=160a5b91371e598957e10178fb3aa143.

[55] Cafiso V., Lo Verde F., Zega A., Pigola G., Rostagno R., Borrè S., Stefani S. (2021). Genomic Characterization of a New Biofilm Forming and Adhesive ST398 Human-Adapted MSSA Lineage Causing Septic Knee Arthritis Following Surgical Reconstruction. Microorganisms.

[56] Cingolani P., Platts A., Wang l., Coon M., Nguyen T., Wang L., Land S.J., Lu X., Ruden D.M. (2012). A program for annotating and predicting the effects of single nucleotide polymorphisms, SnpEff: SNPs in the genome of *Drosophila melanogaster* strain w1118; iso-2; iso-3. Fly.

[57] Zankari E., Hasman H., Cosentino S., Vestergaard M., Rasmussen S., Lund O., Aarestrup F.M., Larsen M.V. (2012). Identification of acquired antimicrobial resistance genes. J. Antimicrob. Chemother..

[58] Bartels M.D., Petersen A., Worning P., Nielsen J.B., Larner-Svensson H., Johansen H.K., Andersen L.P., Jarløv J.O., Boye K., Larsen A.R. (2014). Comparing whole-genome sequencing with Sanger sequencing for spa typing of methicillin-resistant *Staphylococcus aureus*. J. Clin. Microbiol..

[59] Joensen K.G., Scheutz F., Lund O., Hasman H., Kaas R.S., Nielsen E.M., Aarestrup F.M. (2014). Real-time whole-genome sequencing for routine typing, surveillance, and outbreak detection of verotoxigenic *Escherichia coli*. J. Clin. Microbiol..

[60] Carattoli A., Hasman H. (2020). PlasmidFinder and in silico pMLST: Identification and typing of plasmid replicons in whole-genome sequencing (WGS). Horizontal Gene Transfer.

[61] Johansson M., Bortolaia V., Tansirichaiya S., Aarestrup F.M., Roberts A.P., Petersen T.N. (2021). Detection of mobile genetic elements associated with antibiotic resistance in *Salmonella enterica* using a newly developed web tool: MobileElementFinder. J. Antimicrob. Chemother..

[62] Zhou Y., Liang Y., Lynch K.H., Dennis J.J., Wishart D.S. (2011). PHAST: A fast phage search tool. Nucleic Acids. Res..

[63] Van Der Mee-Marquet N., Corvaglia A.R., Valentin A.S., Hernandez D., Bertrand X., Girard M., Kluytmansf J., Donnio P.Y., Francois P. (2013). Analysis of prophages harboured by the human-adapted subpopulation of *Staphylococcus aureus* CC398. Infect. Genet. Evol..

[64] Huang D.W., Sherman B.T., Lempicki R.A. (2009). Systematic and integrative analysis of large gene lists using DAVID bioinformatics resources. Nat. Protoc..

[65] Ramadhan A.A., Hegedus E. (2005). Survivability of vancomycin resistant enterococci and fitness cost of vancomycin resistance acquisition. J. Clin. Pathol..

[66] Cafiso V., Stracquadanio S., Lo Verde F., Dovere V., Zega A., Pigola G., Aranda J., Stefani S. (2020). COLR *Acinetobacter baumannii* sRNA Signatures: Computational Comparative Identification and Biological Targets. Front. Microbiol..

