# Supplementary material for: New Antimicrobial Resistance Strategies: An Adaptive Resistance Network Conferring Reduced Glycopeptide Susceptibility in VISA
Source: Antibiotics (Basel). 2023 Apr 19;12(4):783. doi: 10.3390/antibiotics12040783 (PMC10135003; doi:10.3390/antibiotics12040783)
Supplement: Supplementary file 1 [file antibiotics-12-00783-s001.zip › antibiotics-2298855-supplementary.pdf]

Figure S1. **Validation of *suc*A asRNA**

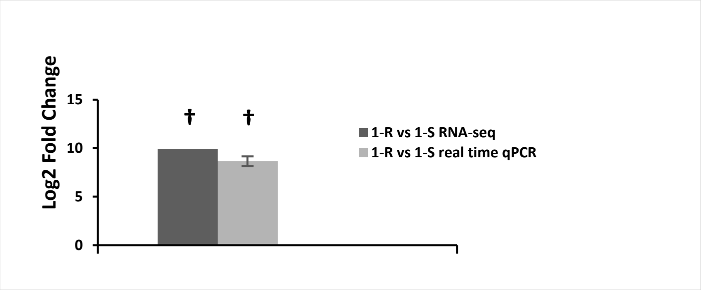

†: Statistically significant difference

Table S1. DEGs and asRNAs in targets putatively related to glycopeptide resistance in VISA CA-MRSA

NAME	SYNON.	PRODUCT	Strand	Transcr. Start	Transl. Start	Transl. Stop	Trans. Stop	asRNA type	RPKM 1S	EXP 1S	RPKM 1R	EXP 1R	qValue	EXP TREND RATE 1R vs 1S
**Cell wall biosynthesis**														
**murF**	**MW2005**	**UDP-N-acetylmuramoylalanyl-D-glutamyl-2, 6-diaminopimelate-D-alanyl-D-alanyl ligase**	**-**		**2166005**	**2164647**			**0**	**0**	**36**	**59**	**0**	**↑**
-	Predicted RNA	Antisense: murF	+	2164976			2164998	cis-asRNA (~3' end)	761	1136	0	0	0	↓↓↓
-	Predicted RNA	Antisense: murT Lipid II D-glutamate to an isoglutamine	+	1994502			1994528	cis-asRNA (internal)	761	1136	0	0	0	↓↓↓
-	Predicted RNA	Antisense: murT Lipid II D-glutamate to an isoglutamine	+	1995323			1995345	cis-asRNA (internal)	761	1136	0	0	0	↓↓↓
**Peptidoglycan aminoacids**														
ddl	MW2006	D-alanyl-alanine synthetase A	-		2167090	2166020			17	25	0	0	0	↓
-	Predicted RNA	Antisense: ddl	+	2166814			2166837	cis-asRNA (~5' end)	1522	2272	0	0	0	↓↓↓
murI	MW1033	Glutamate racemase (L-glutamate in D-glutamate)	+		1132077	1132877			27	41	0	0	0	↓
alr	MW1994	Alanine racemase (L-ala in D-ala)	-		2151144	2149996			22	33	0	0	0	↓
dapD	MW1285	Tetrahydrodipicolinate acetyltransferase (L-lysine biosynthesis via DAP)	+		1405382	1406101			25	37	0	0	0	↓
**-**	**Predicted RNA**	**Antisense: dapE (L-lysine biosynthesis via DAP)**	**-**	**2093354**			**2093327**	**cis-asRNA (~3' end)**	**0**	**0**	**3258**	**5293**	**0**	**↑↑↑**
hutI		hutI (L-lysine) (degradation of L-histidine into L-glutamate)	-		2398595	2397357			27	40	0	0	0	↓
**hutG**		**hutG (L-lysine) (degradation og L-histidine into L-glutamate)**	**-**		**2402728**	**2401793**			**0**	**0**	**49**	**80**	**0**	**↑**
-	Predicted RNA	Antisense: hutH (L-Lysine) (L-histidine degradation into L-glutamate)	-	12338			12310	cis-asRNA (~3' end)	761	1136	0	0	0	↓↓↓
**mqo1**	**MW2286**	**Malate:quinone oxidoreductase Mqo1 (oxalacetate substrate for lysine biosynthesis)**	**-**		**2433407**	**2431872**			**0**	**0**	**28**	**47**	**1,04E-19**	**↑**
-	Predicted RNA	Antisense: mqo2	+	2690445			2690497	cis-asRNA (~3' end)	0	0	736	1196	0	↑↑↑
-	Predicted RNA	Antisense: mqo2	+	2690834			2690887	cis-asRNA (~3' end)	0	0	600	975	8,22E-03	↑↑↑
-	Predicted RNA	Antisense: mqo2	+	2691351			2691488	cis-asRNA (~ 5' end)	0	0	390	634	0	↑↑
**Biosynthesis of the peptigoglycan amino sugar precursor**														
	predicted RNA	antisense: glmU NAG biosynthesis	-	513199			513178	cis-asRNA (internal)	761	1136	0	0	0	↓↓↓
**Biosynthesis TA**														
tarJ	MW0228	tarJ: biosynthesis of poly(ribitol phosphate) teichoic acid	+		271614	272639			36	54	0	0	1,05E-113	↓
mnaA	MW2035	UDP-GlcNAc 2-epimerase (UDP-N-acetylglucosamine (UDP-GlcNAc) in UDP-N-acetylmannosamine (UDP-ManNAc)	-		2190015	2188885			0	0	34	56	0	↑
**-**	**Predicted RNA**	**Antisense: mnaA**	**+**	**2189088**			**2189116**	**cis-asRNA (~3' end)**	**0**	**0**	**1497**	**2432**	**3,50E-15**	**↑↑↑**
-	Predicted RNA	Antisense: mnaA	+	2189792			2189825	cis-asRNA (~5' end)	1522	2272	0	0	0	↓↓↓

-	Predicted RNA	Antisense: ispD (tarI1): biosynthesis of poly(ribitol phosphate)<br>Biosynthesis of teichoic acid	-	277261			277236	cis-asRNA (~3' end)	761	1136	0	0	0	↓↓↓
-	Predicted RNA	antisense: ispD (tarI1) –<br>Poly(ribitol phosphate) teichoic acid biosynthesis	-	277499			277480	cis-asRNA(~3' end)	761	1136	0	0	2,97E-08	↓↓↓
**Biosynthesis of LTA**														
-	Predicted RNA	Antisense: MW0681 lipoteicocic acid synthetase ltaS	-	761881			761850	trans-acting sRNA ltaS (UTR5' and 5' end)	761	1136	0	0	0	↓↓↓
**Charge of the cellenvelope**														
**dltA**	**MW0814**	**D-alanine–poly(phosphoribitol) ligase subunit 1**	**+**		**898360**	**899817**			**0**	**0**	**83**	**135**	**0**	**↑↑**
dltD	MW0817	Poly D-alanine transfer protein (glycerophosphate chain)	+		901279	902454			18	27	0	0	0	↓
**fmtC/mprF**	**MW1247**	**Oxacillin resistance-related FmtC/MprF protein**	**+**		**1365602**	**1368124**			**0**	**0**	**23**	**38**	**0**	**↑**
-	Predicted RNA	Antisense: fmtC/mprF	-	1365760			1365737	cis-asRNA (~ 5' end)	761	1136	0	0	0	↓↓↓
-	Predicted RNA	Antisense: fmtC/mprF	-	1366276			1366249	cis-asRNA (~ 5' end)	1522	2272	0	0	0	↓↓↓
**Autolysins**														
-	Predicted RNA	Antisense: atl	+	1031027			1031048	cis-asRNA (~3' end)	761	1136	0	0	0	↓↓↓
-	Predicted RNA	Antisense: sceD	+	2177015			2177035	cis-asRNA sceD 5'end	761	1136	0	0	0	↓↓↓
**-**	**Predicted RNA**	**Antisense: isaA**	**+**	**2657852**			**2657876**	**cis-asRNA (internal)**	**0**	**0**	**1649**	**2678**	**0**	**↑↑↑**
**-**	**Predicted RNA**	**Antisense: isaA**	**+**	**2658100**			**2658160**	**cis-asRNA (~5' end)**	**0**	**0**	**603**	**980**	**0**	**↑↑↑**
**Cell division**														
ftsY	MW1118	Signal recognition particle FtsY	+		1220509	1221759			12	19	0	0	0	↓
**-**	**Predicted RNA**	**Antisense: ftsL**	**-**	**1157544**			**1157522**	**cis-asRNA(internal)**	**0**	**0**	**1649**	**2678**	**0**	**↑↑↑**
-	Predicted RNA	Antisense: ftsA	-	1164426			1164397	cis-asRNA(~ 3'end)	761	1136	0	0	0	↓↓↓
rodA	MW2007	Cell division protein RodA	+		2167408	2168610			30	46	0	0	1,05E-113	↓
-	Predicted RNA	Antisense: rodA	-	2167562			2167541	cis-asRNA (~5' end)	761	1136	0	0	0	↓↓↓
**-**	**MW2071**	**Lytic regulatory protein**	**+**		**2224956**	**2226047**			**0**	**0**	**32**	**52**	**0**	**↑**
-	Predicted RNA	Antisense: spoVG Putative septation protein SpoVG	-	511742			511715	cis-asRNA (~5' end)	761	1136	0	0	0	↓↓↓
**Transporter**														
**-**	**MW0539**	**MW0539 amino acid permease**	**+**		**624466**	**625950**			**0**	**0**	**37**	**60**	**0**	**↑**
-	MW0585	Putative monovalent cation/H+ antiporter subunit A	+		666582	668984			9	13	0	0	0	↓
-	MW0586	Putative monovalent cation/H+ antiporter subunit B	+		668971	669396			39	58	0	0	0	↓
-	MW0587	Putative monovalent cation/H+ antiporter subunit C	+		669393	669737			57	85	0	0	0	↓
-	MW0588	Putative monovalent cation/H+ antiporter subunit D	+		669727	671223			10	15	0	0	0	↓

aapA	MW1640	D-serine/D-alanine/glycine transporter	+		1771567	1772928			15	22	0	0	0	↓
oppF	MW0181	Oligopeptide transport ATP-binding protein	-		212968	211376			15	23	0	0	0	↓
oppD	MW0870	OppD	+		959996	961078			17	26	0	0	0	↓
tcyC	MW2334	TcyC – AA transporter	-		2483511	2482780			23	35	0	0	0	↓
tcyA	MW2336	TcyA – AA transporter	-		2484987	2484208			0	0	98	160	0	↑↑
-	Predicted RNA	Antisense: MW2336 (tcyA)	+	2484179			2484243	cis-asRNA (~ 5' end)	0	0	651	1057	0	↑↑↑
-	Predicted RNA	Antisense: tcyP	+	405895			405927	cis-asRNA (internal)	0	0	1099	1785	0	↑↑↑
Glycopeptide resistance														
tcaA	MW2277	TcaA	-		2424763	2423381			0	0	33	54	0	↑
tcaB	MW2358	TcaB	+		2706933	2705563			0	15	5	131	0,001178	↑↑
gdpP	MW0014	GdpP	+		18322	20289			10	15	0	0	0	↓
PURINE														
purL	MW0952	Phosphoribosylformylglycinamidine synthase II	+		1046476	1048665			0	0	49	79	0	↑
-	predicted RNA	Antisense: purK	-	1044556			1044512	cis-asRNA (~ 3' end)	0	0	1209	1964	0	↑↑↑
PYRIMIDINE									10	15	0	0	0	
pyrR	MW1081	Bifunctional pyrimidine regulatory protein PyrR uracil phosphoribosyltransferase	+		1177585	1178112			33	49	0	0	0	↓
pyrP	MW1082	Uracil permease	+		1178330	1179637			17	26	0	0	0	↓
pyrAA	MW1085	Carbamoyl phosphate synthase small subunit	+		1178330	1179637			5	143	44	1163	0,002787	↑↑↑
pyrH	MW1141	Uridylate kinase	+		1247297	1248019			23	34	0	0	0	↓
-	predicted RNA	Antisense: pykA	+	1774446			1774466	cis-asRNA (internal)	761	1136	0	0	0	↓↓↓
	predicted RNA	Antisense: pyrG	+	2204490			2204578	cis-asRNA (~ 3' end)	0	0	809	1314	0	↑↑↑
upp	MW2036	Uracil phosphoribosyltransferase	-		2190665	2190036			0	0	94	153	0	↑↑
-	predicted RNA	Antisense: upp	+	2190357			2190384	cis-asRNA (internal)	1386	2069	0	0	0	↓↓↓
REGULATORS														
walR	MW0018	Response regulator walR	+		24930	25631			27	40	0	0	0	↓
walK	MW0019	Two-component sensor histidine kinase walK	+		25644	27470			23	35	0	0	0	↓
-	predicted RNA	Antisense: walH	-	28620			28601	cis-asRNA (internal)	761	1136	0	0	2,97E-08	↓↓↓
-	predicted RNA	Antisense: walJ	-	30497			30475	cis-asRNA (~3' end)	761	1136	0	0	0	↓↓↓
sarH1	MW0085	SarS	-		102180	101428			26	39	0	0	0	↓
-	Predicted RNA	Antisense:sarZ	-	2456896			2456819	cis-asRNA (internal)	0	0	606	984	0	↑↑↑

-	Predicted RNA	Antisense: rpoB	-	570795			570766	cis-asRNA (~5' end)	1522	2272	0	0	0	↓↓↓
-	Predicted RNA	Antisense: rpoB	-	571119			571095	cis-asRNA (internal)	0	0	1561	2535	0	↑↑↑
-	Predicted RNA	Antisense: rpoC	-	577326			577301	cis-asRNA (~3' end)	0	0	2114	3434	0	↑↑↑
-	Predicted RNA	Antisense: vraB	-	619523			619499	cis-asRNA (internal)	0	0	2572	4178	0	↑↑↑
-	Predicted RNA	Antisense: vraB	-	619939			619809	cis-asRNA (internal)	0	0	553	899	0	↑↑
-	Predicted RNA	Antisense: vraB	-	620196			620148	cis-asRNA (~3' end)	0	0	919	1494	0	↑↑↑
-	Predicted RNA	Antisense: vraC vraB	-	620380			620264	Trans-sRNA intergenic region vraC vraB	0	0	404	656	0	↑↑
-	Predicted RNA	Antisense: vraX	+	621196			621257	cis-asRNA (~5' end)	0	0	594	964	0	↑↑↑
graR	MW0621	GraR	+		703600	704274			32	48	0	0	0	↓
-	Predicted RNA	Antisense: vraF	-	706093			706046	cis-asRNA (~3'-end)	0	0	1042	1692	0	↑↑↑
saeR	MW0668	Response regulator saeR	-		752654	751968			0	0	89	145	0	↑↑
-	Predicted RNA	Antisense: saeS	+	750905			750970	cis-asRNA (~3' end)	0	0	2948	4788	0	↑↑↑
-	Predicted RNA	Antisense: saeS	+	751289			751315	cis-asRNA (~5' end)	761	1136	0	0	0	↓↓↓
codY	MW1138	Transcriptional repressor CodY	+		1244215	1244988			39	58	0	0	0	↓
-	Predicted RNA	Antisense: sigA	+	1641370			1641389	cis-asRNA (~3' end)	761	1136	0	0	2,97E-08	↓↓↓
rot	MW1705	Repressor of toxins Rot	-		1860076	1859615			0	0	72	117	1,87E-03	↑↑
vraR	MW1824	Two-component response regulator	-		1987943	1987314	1987314		0	0	232	378	0	↑↑
-	Predicted RNA	Antisense: vraR	+	1987481			1987528	cis-asRNA (internal)	761	1136	0	0	0	↓↓↓
vraS	MW1825	Two-component sensor histidine kinase	-		1988976	1987933			0	0	270	438	0	↑↑
vraT	MW1826	VraT	-	1989688	1989674	1988973			0	0	247	401	0	↑↑
vraU	MW1827	VraU	-		1990075	1989689			0	0	92	149	0	↑↑
hld	MW1959	Delta-hemolysin	-	2107813	2107813	2107679	2107679		1685	2516	0	0	0	↓↓↓
agrB	MW1960	Accessory gene regulator B	+		2108049	2108612			130	195	0	0	0	↓↓
agrC	MW1962	AgrC	+		2108781	2110073			8	212	0	0	0	↓
agrA	MW1963	AgrA	+	2110092	2110092	2110808	2110808		27	717	0	11	2,57E-57	↓
-	Predicted RNA	Antisense: agrB	-	2108052			2108025	trans-acting agrB	6794	10146	0	0	0	↓↓↓
-	Predicted RNA	Antisense: agrB	-	2108168			2108132	cis-asRNA (~5'-e d)	1522	2272	0	0	0	↓↓↓
-	Predicted RNA	Antisense: agrB	-	2108441			2108405	cis-asRNA (internal)	987	1474	0	0	0	↓↓↓
-	Predicted RNA	Antisense: agrD	-	2108703			2108652	cis-asRNA (~3' end)	2883	4305	0	0	0	↓↓↓
-	Predicted RNA	Antisense: agrC	-	2108870			2108826	cis-asRNA (internal)	761	1136	0	0	6,92E-94	↓↓↓

-	Predicted RNA	Antisense: agrC	-	2109347			2109286	cis-asRNA (internal)	1595	2382	0	0	0	↓↓↓
-	Predicted RNA	Antisense: agrC	-	2109526			2109477	cis-asRNA (internal)	2283	3409	0	0	0	↓↓↓
-	Predicted RNA	Antisense: agrC	-	2109667			2109635	cis-asRNA (internal)	1475	2203	0	0	0	↓↓↓
-	Predicted RNA	Antisense: agrC	-	2109822			2109768	cis-asRNA (intrernal)	1784	2665	0	0	0	↓↓↓
-	Predicted RNA	Antisense: agrC	-	2110013			2109950	cis-asRNA (~3' end)	927	1384	0	0	0	↓↓↓
-	Predicted RNA	Antisense: agrA	-	2110216			2110191	cis-asRNA (~5' end))	761	1136	0	0	0	↓↓↓
-	Predicted RNA	Antisense: agrA	-	2110451			2110304	cis-asRNA (internal)	7666	11448	81	132	1,06E-82	↓↓↓
-	Predicted RNA	Antisense: agrA	-	2110603			2110531	cis-asRNA (internal)	500	747	0	0	0	↓↓
-P	Predicted RNA	Antisense: agrA	-	2110719			2110696		1426	2130	0	0	6,92E-94	↓↓↓

Legend. Red, DE coding genes; black, DE antisense small RNA; bold, overexpression trend.

Differential expression rate: ↑/↓-low, ↑↑/↓↓-medium, ↑↑↑/↓↓↓-high differential expression

Table S2. Comparative Analysis of the nsSNPs in intergenic genomic regions in 1R vs. 1S.

Genomic Localization	Nucleotide	nsSNPs	Locus Tag	Effect Prediction
Intergenic region	636844	A>C	MW0550-MW0551	Modifier
Intergenic region	648981	T>G	MW0564-MW0565	Modifier
Intergenic region	825243	C>A	*rsa*H-MW0732	Modifier
Intergenic region	919233	A>T	MW0836-MW0837	Modifier
Intergenic region	940658	T>C	MW0850-MW0851	Modifier
Intergenic region	1209748	A>T	MW1109-*rec*G	Modifier
Intergenic region	2202656	G>T	*mur*Z-*fba*A	Modifier
Intergenic region	2377514	T>C	MW2229-MW2230	Modifier
